# Prevalence and associated factors of anxiety in postpartum women

**DOI:** 10.18332/ejm/204308

**Published:** 2025-06-12

**Authors:** María Antonia Díaz Ogallar, Sergio Martínez Vázquez, Antonio Hernández Martínez, Rocío Adriana Peinado Molina, Juan Miguel Martínez Galiano

**Affiliations:** 1 Department of Nursing, University of Jaen, Jaen, Spain; 2 Department of Nursing, Faculty of Nursing of Ciudad Real, University of Castilla-La Mancha, Ciudad Real, Spain; 3 Consortium for Biomedical Research in Epidemiology and Public Health, Madrid, Spain

**Keywords:** prevalence, associated factors, anxiety, postpartum

## Abstract

**INTRODUCTION:**

Postpartum anxiety affects approximately 1 in 4 women, with significant implications for both mothers and newborns. This study aimed to identify factors associated with postpartum anxiety and to assess its prevalence.

**METHODS:**

A cross-sectional study was carried out with women in the postpartum period in Spain. To measure attachment (VAMF bonding and VAMF attachment), the Maternal-Child Bond and Attachment - VAMF tool was used. Purpose sampling method was used to collect data from postpartum and postnatal consultations in medical centers. Sociodemographic and obstetric variables, anxiety level (GAD-7), risk of intimate partner violence (WAST), and risk of postpartum depression (EPDS) were obtained. Odds ratios (ORs) and adjusted odds ratios (AORs) with their respective 95% confidence intervals (CIs) were calculated.

**RESULTS:**

A total of 820 women participated, 36.1% (296) experienced mild anxiety, 8.5% (70) moderate anxiety, and 2.7% (22) severe anxiety. Key risk factors for anxiety included higher EPDS scores (adjusted odds ratio, AOR=1.68; 95% CI: 1.55–1.81), smoking (AOR=1.97; 95% CI: 1.01–3.82), a history of mental health issues (AOR=1.77; 95% CI: 1.13–2.79), and challenges related to the baby's health (AOR=2.70; 95% CI: 1.34–5.47). Additionally, a high-risk score on the WAST was linked to increased anxiety (AOR=1.53; 95% CI: 1.01–2.31). Conversely, protective factors included a positive mother–baby bonding score (AOR=0.90; 95% CI: 0.85–0.96) and a monthly income of 1000–1999 € (AOR=0.55; 95% CI: 0.31–0.95). For moderate to severe anxiety, a high EPDS score remained a notable risk factor (AOR=1.35; 95% CI: 1.26–1.44), while positive bonding (AOR=0.92; 95% CI: 0.85–0.98), higher income (>2000 €) (AOR=0.35; 95% CI: 0.15–0.80), and favorable treatment by healthcare providers significantly reduced anxiety risk (AOR=0.21; 95% CI: 0.07–0.70).

**CONCLUSIONS:**

The prevalence of anxiety in women after childbirth is high. Depression, poor bonding, and economic status are factors that influence the onset of postpartum anxiety.

## INTRODUCTION

When women become mothers, they are under tremendous pressure during the perinatal period, leading to anxiety and stress due to prevailing role models and social expectations^1^. The perinatal period is considered a critical phase in a woman’s life, and is not without difficulties for mental health^2^. In particular, the postpartum period can be emotionally challenging for mothers, with high levels of anxiety, stress, and depression.

Anxiety manifests itself through agitation, palpitations, nausea, control problems, irrational thoughts, or social avoidance^3^. Anxiety can begin to be experienced by a mother during pregnancy and up to the first year postpartum and is referred to as postpartum anxiety^3^.

Although postpartum anxiety is an underdiagnosed problem^1^, it can be present in up to 42% of women after giving birth^4-6^. This figure varies depending on severity and other factors, but globally, it is estimated that 1 in 4 women will experience postpartum anxiety, which can last up to a year after childbirth^4^.

Although the causes associated with the appearance of anxiety are varied, the literature has described socio-economic factors^7^, low social support^8^, obstetric variables such as the experience of childbirth and the type of birth, and even previous mental health disorders of the woman herself ^9,10^. Cohabitation with the partner and the couples’ relationship has also been described as a variable associated with the onset of postpartum anxiety^11^.

Anxiety, to whatever degree, has been associated with an increased likelihood of developing adverse outcomes in both mother and newborn^12,13^. Thus, in the postpartum period, anxiety can influence the attachment between the baby and the mother^14^. It can also affect the social and occupational level in the late postpartum period^15^.

Women who suffer from anxiety after childbirth may develop other disorders during the postpartum stage, such as postpartum depression^7,12,16^, maternal stress^11^ or suicidal ideation, which is the main predictor of perinatal suicide^17,18^. It has been estimated that anxiety-associated comorbidity costs an estimated £8.1 billion per birth cohort per year in countries such as the UK^19^.

Considering the magnitude and impact of postpartum anxiety on maternal and neonatal health, together with the few studies that address this pathology (most studies focus on postpartum depression), as well as the recommendation to carry out studies on postpartum anxiety^20^, the present study aims to study the factors associated with the presence of this postpartum anxiety, as well as to determine the prevalence of this disorder.

## METHODS

### Design and participant selection

A cross-sectional study was conducted on women who had given birth in Spain during the last half of 2021 and the first half of 2022 who met the following inclusion criteria: having given birth less than 18 months ago and not having suffered a neonatal loss. Women under 18 years of age and those who did not understand Spanish (language barrier) were excluded.

In an attempt to estimate the sample size to obtain valid estimations, the maximum modeling principle^21^ was followed. This requires 10 events (women with anxiety) per each included variable. Considering that the prevalence in this reference population and sociodemographic context ranges 20–25%^5^, it would be necessary to recruit a sample of 800 women (200 with anxiety) for an initial model of 20 variables.

The questionnaire was distributed by collaborating health workers in the clinical setting, including during postpartum and postnatal consultations, as well as midwifery consultations in medical centers (including hospital or health center as well as midwife-led clinics). This allowed more women to be recruited (via purpose sampling method) including during the subsequent check-ups where the mother attended with the newborn. Once women were selected, they were given the information about the study and lately the choice to participate and sign the informed consent form. Mechanisms for resolving queries were established through a WhatsApp group among collaborating professionals in order to provide homogeneous answers to any questions that might arise.

### Ethical considerations

The present study was approved by the Research Ethics Committee of the Province of Jaén (DCVA-21/2012-N-21). All women participating in the study signed the informed consent form.

### Information source and study variables

The data were collected using a self-developed, previously piloted questionnaire, which was distributed in different hospitals and health centers. This questionnaire contained open and closed questions, with a language understandable to all educational levels. It included sociodemographic variables such as age, income level, lifestyles such as alcohol or tobacco consumption, obstetric, family, and personal history of the pregnancy itself, and also variables related to the newborn. To assess the presence of anxiety, the Generalized Anxiety Disorder Screener (GAD-7) scale was used in its validated version in a population similar to that of our study^20^. The GAD-7 is a self-administered 7-item scale with four response options in ascending order from 0 to 4 points (never, several days, half of the days, almost every day), with a score ≥5 indicating the presence of anxiety and ≥10 indicating moderate or severe anxiety^20^.

To measure mother-child bond and attachment, the "Maternal - Child Bond and Attachment" ("VAMF", for its initials in Spanish) tool was used. The VAMF tool is a self-administered 29-item scale that measures mother-child bonding and attachment, and is designed and validated for application in the postpartum period and up to 18 months of age of the infant. The Edinburgh Postpartum Depression Scale (EPDS)^23^ was used to determine the risk of postpartum depression (PPD). The Woman Abuse Screening Tool (WAST) was used to screen for Intimate Partner Violence (IVP)^24^. All these instruments have been validated in a population similar to that of the present study.

### Statistical analysis

For sociodemographic and clinical data, absolute (n) and relative frequencies (%) were used to describe qualitative variables, and mean and standard deviation (SD) were used to describe quantitative variables. A bivariate and multivariate analysis was then performed between anxiety risk and moderate-severe anxiety risk with possible associated factors. In the first case, anxiety risk was considered as ‘No anxiety’ with scores <5 on the GAD-7 and ‘Anxiety’ with scores ≥5. In the case of moderate-severe anxiety risk, the comparison was between ‘Low or no anxiety levels’ with scores <10 and ‘Moderate-severe anxiety’ with scores ≥10.

Binary logistic regression was used to estimate odds ratios (ORs) and adjusted odds ratios (AORs) with their respective 95% confidence intervals. When multivariate analysis was performed, the backward stepwise procedure was used, and all variables from the bivariate analysis were included whether or not statistical significance was observed. For this analysis, the dependent variable GAD-7 score was dichotomized, while the independent variables were entered into the model in their original form (age, the VAMF and EPDS scales continuously and without transformations or categorizations).

Finally, the predictive ability of the model was estimated using the area under the receiver operating characteristic (ROC) curve (AUC). In order to assess the prediction in qualitative terms, the Swets criterion^25^ was used with values: 0.5–0.6 (bad), 0.6–0.7 (poor), 0.7–0.8 (satisfactory), 0.8–0.9 (good), and 0.9–1.0 (excellent). The statistical program SPSS 29.0 was used for data analysis.

## RESULTS

A cross-sectional study was conducted with 820 postpartum women in Spain. The mean age was 34.30 years (SD=4.06), 58.7% (481) were primiparous and 41.2% (338) were multiparous; 9.0% (47) of the women had an unplanned pregnancy, while 13.5% (111) needed fertility treatment. A total of 24.9% (204) had experienced some mental issues during their lifetime. In terms of experience during labor, 11.5% (94) had a bad experience, and 70.4% (577) defined it as good or very good. The treatment received by the professional healthcare team was defined as bad or very bad by 3.4% (28) women, while 88.9% (729) described it as good or very good; 33.8% (277) of the women were screened as high-risk from suffering IPV. The mean score of EPDS was 7.44 (SD=4.70). Related to anxiety, 52.7% (423) of the women did not suffer any kind of anxiety, whereas 36.1% (296) suffered from mild anxiety, 8.5% (70) moderate, and 2.7% (22) severe anxiety. The mean score of anxiety in the whole sample was 4.94 (SD=3.83). The rest of the data can be seen in Table 1.

**Table 1:** Sociodemographic and clinical characteristics of the study sample, Spain, 2021–2022 (N=820)

*Variable*	*Mean (SD)*
**Age** (years)	34.30 (4.06)
**EPDS**	7.44 (4.70)
**VAMF bonding**	57.81 (3.91)
**VAMF attachment**	42.72 (4.87)
**GAD-7**	4.94 (3.83)
***Variable***	***n (%)***
**Anxiety level**	
No anxiety	432 (52.7)
Mild	296 (36.1)
Moderate	70 (8.5)
Severe	22 (2.7)
**Income level** (€)	
<1000	140 (17.1)
1000–1999	444 (54.1)
>2000	236 (28.8)
**Alcohol consumption**	
Never	272 (33.2)
Occasionally	492 (60.0)
Frequently	56 (6.8)
**Smoking habit**	
No	740 (90.2)
Yes	80 (9.8)
**Number of children**	
1	599 (73.0)
2	191 (23.3)
≥3	30 (3.7)
**Pregnancy**	
1	481 (58.7)
2	222 (27.1)
≥3	116 (14.1)
Missing	1 (0.1)
**Planned pregnancy**	
No	74 (9.0)
Yes	746 (91.0)
**Cesarean birth**	
No	608 (74.1)
Yes	212 (25.9)
**Fertility treatment** (IVF, egg donation, etc.)	
No	709 (86.5)
Yes	111 (13.5)
**Depression** (current)	
No	741 (90.4)
Yes	79 (9.6)
**Antenatal classes**	
No	217 (26.5)
Yes	603 (73.5)
**High risk pregnancy**	
No	695 (84.8)
Yes	125 (15.2)
**Any illness** (current)	
No	721 (87.9)
Yes	99 (12.1)
**Mental health issues** (any time during life)	
No	616 (75.1)
Yes	204 (24.9)
**Feeling tired during pregnancy, labor or postpartum**	
No	85 (10.4)
Yes	735 (89.6)
**Type of birth**	
Vaginal	484 (59.0)
Instrumental	158 (19.3)
Cesarean section (elective)	45 (5.5)
Cesarean section (emergency)	133 (16.2)
**Admission to ICU**	
No	809 (98.7)
Yes	11 (1.3)
**Hospital readmission**	
No	802 (97.8)
Yes	18 (2.2)
**Baby admission to pediatrics unit**	
No	734 (89.5)
Yes	67 (8.2)
Yes, NICU admission	19 (2.3)
**Skin-to-Skin**	
No	123 (15.0)
Yes	697 (85.0)
**Preterm baby**	
No	782 (95.4)
Yes	38 (4.6)
**Baby with problem** (current)	
No	740 (90.2)
Yes	80 (9.8)
**Currently breastfeeding**	
No	98 (12.0)
Yes	722 (88.0)
**Experience during labor**	
Bad or very bad	94 (11.5)
Not sure	149 (18.2)
Good or very good	577 (70.4)
**Experience** (professional treatment)	
Bad or very bad	28 (3.4)
Not sure	63 (7.7)
Good or very good	729 (88.9)
**Support received from family**	
Low or very low	69 (8.4)
Moderate	160 (19.5)
High or very high	591 (72.1)
**WAST** (IPV Risk)	
Low risk	543 (66.2)
High risk	277 (33.8)

The relation between the different scales was studied, finding a statistically significant relation between the mean score of EPDS (p≤0.001), VAMF bonding (p≤0.001), and VAMF attachment (p=0.002), and the presence of any kind of anxiety. This can be seen in Table 2.

**Table 2:** Distribution of scores between EPDS, VAMF bonding, VAMF attachment, and the presence of anxiety, Spain, 2021–2022 (N=820)

*Variable*	*Anxiety*			
	*No* *mean(SD)* *(N=432)*	*Yes* *mean (SD)* *(N=388)*	*Mean difference* *(95% CI)*	*p*
**Age** (years)	34.37 (4.28)	34.21 (3.79)	0.16 (-3.98–0.72)	0.079
**EPDS**	4.61 (2.81)	10.59 (4.35)	**-5.98 (-6.48 – -5.49)**	**≤0.001**
**VAMF bonding**	59.01 (2.89)	56.47 (4.44)	**2.54 (2.03–3.04)**	**≤0.001**
**VAMF attachment**	43.06 (4.48)	42.34 (5.25)	**0.72 (0.05–1.39)**	**0.002**

**Figure 1:**
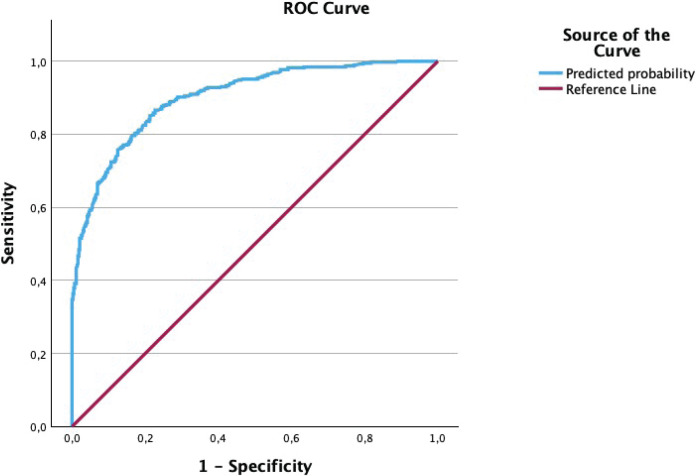
ROC curve for predictive capability for anxiety risk

Subsequently, bivariate and multivariate analyses were performed to determine which factors were associated with any kind of anxiety on the GAD-7 questionnaire compared to no anxiety. Factors that were associated with a higher GAD-7 score were a high EPDS score (AOR=1.68; 95% CI: 1.55–1.81), smoking habit (AOR=1.97; 95% CI: 1.01–3.82), had suffered from any mental issues during lifetime (AOR=1.77; 95% CI: 1.13–2.79), baby having any problem currently (AOR=2.70; 95% CI: 1.34–5.47), or WAST screen positive as high-risk (AOR=1.53; 95% CI: 1.01–2.31). Protective factors associated with anxiety included the VAMF bonding score (AOR=0.90; 95% CI: 0.85–0.96) and a monthly income level between 1000–1999 € (AOR=0.55; 95% CI: 0.31–0.95) appeared. This can be seen in Table 3. The predictive capability for anxiety risk presented a AUC-ROC of 0.90 (95% CI: 0.88– 0.92), with an exceptional capability to classify subjects according to the Swets criterion. The ROC curve can be seen in Figure 1.

**Table 3:** Bivariable and multivariate analysis of the factors associated with anxiety compared to no anxiety, on the GAD-7 questionnaire, Spain, 2021–2022 (N=820)

	*Anxiety*			
	*No* *(GAD-7 <5)*	*Yes* *(GAD-7 ≥5)*	*OR (95% CI)*	*AOR (95% CI)*
***Variable***	***Mean (SD)***	***Mean (SD)***		
**Age** (years)	34.37 (4.28)	34.2 (3.79)	0.99 (0.96–1.02)	
**EPDS**	4.61 (2.81)	10.59 (4.35)	**1.69 (1.57–1.81)**	**1.68 (1.55–1.81)**
**VAMF bonding**	59.01 (2.89)	56.47 (4.44)	**0.82 (0.79–0.86)**	**0.90 (0.85–0.96)**
**VAMF attachment**	43.06 (4.48)	42.34 (5.25)	**0.97 (0.94–0.99)**	
***Variable***	***n (%)***	***n (%)***		
**Income level** (€)				
<1000 ®	62 (44.3)	78 (55.7)	1	1
1000–1999	235 (52.9)	209 (47.1)	0.71 (0.48–1.04)	**0.55 (0.31–0.95)**
>2000	135 (57.2)	101 (42.8)	**0.60 (0.39–0.91)**	0.54 (0.29–0.99)
**Alcohol consumption**				
Never ®	141 (51.8)	131 (48.2)	1	
Occasionally	260 (52.8)	232 (47.2)	0.96 (0.71–1.29)	
Frequently	31 (55.4)	25 (44.6)	0.87 (0.49–1.55)	
**Smoking habit**				
No ®	395 (53.4)	345 (46.6)	1	1
Yes	37 (46.3)	43 (53.8)	1.33 (0.84–2.11)	**1.97 (1.01–3.82)**
**Number of children**				
1 ®	324 (54.1)	275 (45.9)	1	
2	90 (47.1)	101 (52.9)	1.32 (0.95–1.83)	
≥3	18 (60.0)	12 (40.0)	0.79 (0.37–1.66)	
**Pregnancies**				
1 ®	253 (52.6)	228 (47.4)	1	
2	118 (53.2)	104 (46.8)	0.98 (0.71–1.35)	
≥3	61 (52.6)	55 (47.4)	1.00 (0.67–1.50)	
**Planned pregnancy**				
No ®	25 (33.8)	49 (66.2)	1	1
Yes	407 (54.6)	339 (45.4)	**0.43 (0.26–0.70)**	0.54 (0.26–1.09)
**Cesarean birth** (previous)				
No ®	332 (54.6)	276 (45.4)	1	
Yes	100 (47.2)	112 (52.8)	1.35 (0.99–1.84)	
**Fertility treatment** (IVF, egg donation, etc.)				
No ®	372 (52.5)	337 (47.5)	1	
Yes	60 (54.1)	51 (45.9)	0.94 (0.63–1.40)	
**Depression** (current)				
No ®	407 (54.9)	334 (45.1)	1	
Yes	25 (31.6)	54 (68.4)	2.63 (1.60–4.32)	
**Antenatal classes**				
No ®	111 (51.2)	106 (48.8)	1	
Yes	321 (53.2)	282 (46.8)	0.92 (0.67–1.26)	
**High-risk pregnancy**				
No ®	364 (52.4)	331 (47.6)	1	
Yes	68 (54.4)	57 (45.6)	0.92 (0.63–1.35)	
**Any illness** (current)				
No ®	388 (53.8)	333 (46.2)	1	
Yes	44 (44.4)	55 (55.6)	1.46 (0.95–2.22)	
**Mental health issues** (any time during life)				
No ®	360 (58.4)	256 (41.6)	1	1
Yes	72 (35.3)	132 (64.7)	**2.58 (1.86–3.58)**	**1.77 (1.13–2.79)**
**Feeling tired during pregnancy, labor or postpartum**				
No ®	70 (82.4)	15 (17.6)	1	
Yes	362 (49.3)	373 (50.7)	**4.81 (2.70–8.56)**	
**Type of birth**				
Vaginal ®	264 (54.5)	220 (45.5)	1	
Instrumental	86 (54.5)	72 (45.6)	1.01 (0.70–1.44)	
Cesarean section (elective)	22 (48.9)	23 (51.1)	1.26 (0.68–2.31)	
Cesarean section (emergency)	60 (45.1)	73 (54.9)	1.46 (0.99–2.15)	
**Admission to ICU**				
No ®	427 (52.8)	382 (47.2)	1	
Yes	5 (45.5)	6 (54.5)	1.34 (0.41–4.43)	
**Hospital readmission**				
No ®	420 (52.4)	382 (47.6)	1	
Yes	12 (66.7)	6 (33.3)	0.55 (0.20–1.48)	
**Baby admission to pediatrics unit**				
No ®	390 (53.1)	344 (46.9)	1	
Yes	31 (46.3)	36 (53.7)	1.32 (0.80–2.17)	
Yes, NICU admission	11 (57.9)	8 (42.1)	0.83 (0.33–2.07)	
**Skin to Skin**				
No ®	52 (42.3)	71 (57.7)	1	
Yes	380 (54.5)	317 (45.5)	**0.61 (0.42–0.90)**	
**Preterm baby**				
No ®	413 (52.8)	369 (47.2)	1	
Yes	19 (50.0)	19 (50.0)	1.12 (0.58–2.15)	
**Early BF** (1st hour)				
No ®	74 (44.8)	91 (55.2)	1	
Yes	358 (54.7)	397 (45.3)	**0.68 (0.48–0.95)**	
**Currently BF**				
No ®	47 (48.0)	51 (52.0)	1	
Yes	385 (53.3)	337 (46.7)	0.81 (0.53–1.23)	
**Baby with problem** (current)				
No ®	406 (54.9)	334 (45.1)	1	1
Yes	26 (32.5)	54 (67.5)	**2.52 (1.55–4.12)**	**2.70 (1.34–5.47)**
**Experience during labor**				
Bad or very bad ®	35 (37.2)	59 (62.8)	1	
Not sure	73 (49.0)	76 (51.0)	0.62 (0.37–1.05)	
Good or very good	324 (56.2)	253 (43.8)	**0.46 (0.30–0.73)**	
**Experience** (professional treatment)				
Bad or very bad ®	12 (42.9)	16 (57.1)	1	
Not sure	26 (41.3)	37 (58.7)	1.07 (0.43–2.63)	
Good or very good	394 (54.0)	335 (46.0)	0.64 (0.30–1.37)	
**Support received from family**				
Low or very low ®	22 (31.9)	47 (68.1)	1	
Moderate	75 (46.9)	85 (53.1)	**0.53 (0.29–0.96)**	
High or very high	335 (56.7)	256 (43.3)	**0.36 (0.21–0.61)**	
**WAST** (IPV risk)				
Low risk ®	326 (60.0)	217 (40.0)	1	1
High risk	106 (38.3)	171 (61.7)	**2.42 (1.80–3.26)**	**1.53 (1.01–2.31)**

The backward stepwise procedure was used, and all variables from the bivariate analysis were included whether or not statistical significance was observed. For this analysis, the dependent variable GAD-7 was dichotomized, while the independent variables were entered into the model in their original form (age, the VAMF and EPDS scales continuously and without transformations or categorizations). ® reference categories.

**Figure 2:**
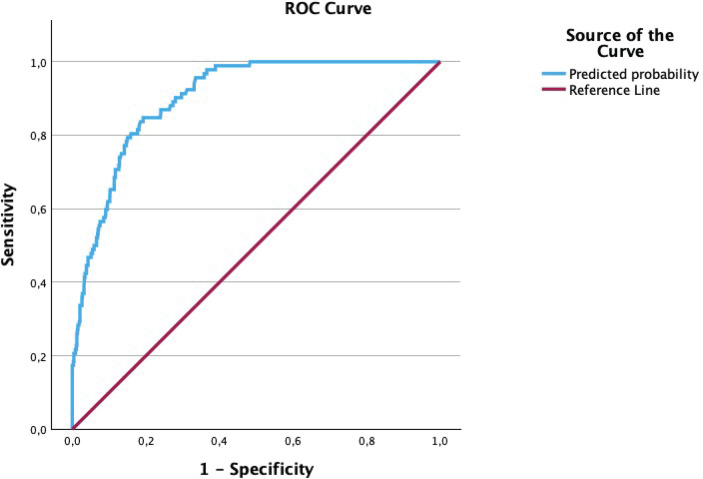
ROC curve for predictive capability for moderate-severe anxiety risk

When we compared no anxiety or mild anxiety and the presence of moderate or severe anxiety by performing bivariate and multivariate analyses to determine which factors were associated with these categories, a high EPDS score (AOR=1.35; 95% CI: 1.26–1.44) appeared as a risk factor. However, VAMF bonding score (AOR=0.92; 95% CI: 0.85–0.98), a monthly income level >2000 € (AOR=0.35; 95% CI: 0.15–0.80), and feeling well treated by healthcare professionals (AOR=0.21; 95% CI: 0.07–0.70) emerged as protective factors. The rest of the results of these analyses can be seen in Table 4. The predictive capability for moderate-severe anxiety risk had a AUC-ROC of 0.90 (95% CI: 0.88–0.93), with an exceptional capability to classify subjects according to the Swets criterion. The ROC curve can be seen in Figure 2.

**Table 4:** Bivariable and multivariate analysis of the factors associated with no or mild anxiety and the presence of moderate or severe anxiety, on the GAD-7 questionnaire, Spain, 2021–2022 (N=820)

	*Anxiety*			
	*No/mild* *(GAD-7 <10)*	*Moderate/severe* *(GAD-7 ≥10)*	*OR (95% CI)*	*AOR (95% CI)*
***Variable***	***Mean (SD)***	***Mean (SD)***		
**Age** (years)	34.30 (4.03)	34.24 (4.28)	1.00 (0.94–1.05)	
**EPDS**	6.64 (4.03)	13.76 (4.84)	**1.39 (1.31–1.47)**	**1.35 (1.26–1.44)**
**VAMF bonding**	58.22 (3.48)	54.59 (5.41)	**0.82 (0.78–0.87)**	**0.92 (0.85–0.98)**
**VAMF attachment**	42.80 (4.74)	42.05 (5.78)	0.97 (0.93–1.01)	
***Variable***	***n (%)***	***n (%)***		
**Income level** (€)				
<1000 ®	116 (82.9)	24 (17.1)	1	1
1000–1999	395 (89.0)	49 (11.0)	0.60 (0.35–1.02)	0.53 (0.27–1.04)
≥2000	217 (91.9)	19 (8.1)	**0.42 (0.22–0.81)**	**0.35 (0.15–0.80)**
**Alcohol consumption**				
Never ®	235 (86.4)	37 (13.6)	1	
Occasionally	443 (90.0)	49 (10.0)	0.70 (0.45–1.11)	
Frequently	50 (89.3)	6 (10.7)	0.76 (0.31–1.90)	
**Smoking habit**				
No ®	662 (89.5)	78 (10.5)	1	1
Yes	66 (82.5)	14 (17.5)	1.80 (0.97–3.36)	2.12 (0.97–5.05)
**Number of children**				
1 ®	538 (89.8)	61 (10.2)	1	
2	162 (84.8)	29 (15.2)	1.58 (0.98–2.54)	
≥3	28 (93.3)	2 (6.7)	0.63 (0.15–2.71)	
**Pregnancies**				
1 ®	426 (88.6)	55 (11.4)	1	
2	199 (89.6)	23 (10.4)	0.90 (0.54–1.50)	
≥3	102 (87.9)	14 (12.1)	1.06 (0.57–1.99)	
**Planned pregnancy**				
No ®	62 (83.8)	12 (16.2)	1	
Yes	666 (89.3)	80 (10.7)	0.62 (0.32–1.20)	
**Cesarean birth** (previous)				
No ®	546 (89.8)	62 (10.2)	1	
Yes	182 (85.8)	30 (14.2)	1.45 (0.91–2.32)	
**Fertility treatment** (IVF, egg donation,				
etc.)				
No ®	630 (88.9)	79 (11.1)	1	
Yes	98 (88.3)	13 (11.7)	1.06 (0.57–1.97)	
**Depression** (current)				
No ®	668 (90.1)	73 (9.9)	1	
Yes	60 (75.9)	19 (24.1)	**2.90 (1.64–5.12)**	
**Antenatal classes**				
No ®	194 (89.4)	23 (10.6)	1	
Yes	534 (88.6)	69 (11.4)	1.09 (0.66–1.80)	
**High-risk pregnancy**				
No ®	617 (88.8)	78 (11.2)	1	
Yes	111 (88.8)	14 (11.2)	1.00 (0.55–1.83)	
**Any illness** (current)				
No ®	649 (90.0)	72 (10.0)	1	
Yes	79 (79.8)	20 (20.2)	**2.28 (1.32–3.95)**	
**Mental health issues** (any time during life)				
No ®	562 (91.2)	54 (8.8)	1	
Yes	166 (81.4)	38 (18.6)	**2.38 (1.52–3.74)**	
**Feeling tired during pregnancy, labor or postpartum**				
No ®	85 (100.0)	0 (0.0)	1	
Yes	643 (87.5)	92 (12.5)	Not calculated	
**Type of birth**				
Vaginal ®	436 (90.1)	48 (9.9)	1	
Instrumental	140 (88.6)	18 (11.4)	1.17 (0.66–2.07)	
Cesarean section (elective)	41 (91.1)	4 (8.9)	0.89 (0.30–2.58)	
Cesarean section (emergency)	111 (83.5)	22 (16.5)	**1.80 (1.04–3.11)**	
**Admission to ICU**				
No ®	720 (89.0)	89 (11.0)	1	
Yes	8 (72.7)	3 (27.3)	3.03 (0.79–11.65)	
**Hospital readmission**				
No ®	714 (89.0)	88 (11.0)	1	
Yes	14 (77.8)	4 (22.2)	2.32 (0.75–7.20)	
**Baby admission to pediatrics unit**				
No ®	651 (88.7)	83 (11.3)	1	
Yes	60 (89.6)	7 (10.4)	0.92 (0.41–2.07)	
Yes, NICU admission	17 (89.5)	2 (10.5)	0.92 (0.21–4.07)	
**Skin to Skin**				
No ®	99 (80.5)	24 (19.5)	1	
Yes	629 (90.2)	68 (9.8)	**0.45 (0.27–0.74)**	
**Preterm baby**				
No ®	695 (88.9)	87 (11.1)	1	
Yes	33 (86.8)	5 (13.2)	1.21 (0.46–3.18)	
**Early BF** (1st hour)				
No ®	139 (84.2)	26 (15.8)	1	
Yes	589 (89.9)	66 (10.1)	**0.60 (0.37–0.98)**	
**Currently BF**				
No ®	81 (82.7)	17 (17.3)	1	
Yes	647 (89.6)	75 (10.4)	**0.55 (0.31–0.98)**	
**Baby with problem** (current)				
No ®	665 (89.9)	75 (10.1)	1	
Yes	63 (78.8)	17 (21.3)	**2.39 (1.33–4.30)**	
**Experience during labor**				
Bad or very bad ®	70 (74.5)	24 (25.5)	1	1
Not sure	134 (89.9)	15 (10.1)	**0.33 (0.16–0.66)**	0.43 (0.17–1.09)
Good or very good	524 (90.8)	53 (9.2)	**0.30 (0.17–0.51)**	1.22 (0.52–2.82)
**Experience** (professional treatment)				
Bad or very bad ®	20 (71.4)	8 (28.6)	1	1
Not sure	48 (76.2)	15 (23.8)	0.78 (0.29–2.13)	0.68 (0.19–2.46)
Good or very good	660 (90.5)	69 (9.5)	**0.26 (0.11–0.62)**	**0.21 (0.07–0.70)**
**Support received from family**				
Low or very low ®	50 (72.5)	19 (27.5)	1	
Moderate	140 (87.5)	20 (12.5)	**0.38 (0.19–0.76)**	
High or very high	538 (91.0)	53 (9.0)	**0.26 (0.14–0.47)**	
**WAST** (IPV risk)				
Low risk ®	496 (91.3)	47 (8.7)	1	
High risk	232 (83.8)	45 (16.2)	**2.05 (1.32–3.17)**	

The backward stepwise procedure was used, and all variables from the bivariate analysis were included whether or not statistical significance was observed. For this analysis, the dependent variable GAD-7 was dichotomized, while the independent variables were entered into the model in their original form (age, the VAMF and EPDS scales continuously and without transformations or categorizations). ® Reference categories.

## DISCUSSION

The study found that nearly 50% of women experienced some level of anxiety postpartum, with over 30% reported mild anxiety, 8% moderate, and nearly 3% severe anxiety. Factors increasing the likelihood of anxiety included higher EPDS scores, smoking, a history of mental health issues, positive WAST results, and having a baby with current problems. In contrast, protective factors against anxiety included strong bonding (VAMF), feeling well-treated by healthcare professionals, and having a moderate to high monthly income, particularly for moderate or severe anxiety.

The prevalence of anxiety detected in our study is slightly higher than that found by other studies, where the prevalence does not exceed 42%^4-6^. Fawcett et al.^5^ found in their systematic review with meta-analysis that 2 in 10 women suffer from postpartum anxiety. Likewise, Dennis et al.^26^, who differentiated between symptoms and the presence of an anxiety diagnosis, also found a lower prevalence than in our population and coincides with those of other authors^4^. This may be due to the tools used. The GAD-7, which has been used in this study, represents a tool that is easy to administer to establish a rapid screening for anxiety in perinatal populations^27^.

Women with higher EPDS scores suffer more frequently from postpartum anxiety, which is in line with different studies^7,12,16^. Sit et al.^12^ found in their study with 628 women, that at least 1 in 2 women has depression and also anxiety as a second diagnosis. Similarly, 41% of women with anxiety had depressive disorders as a second diagnosis. Both pathologies can coexist in such a way that the presence of one of them favors the development of the other^28-30^. Anxiety remains one of the most important risk factors for postpartum depression, highlighting the need for effective screening for its possible presence at multiple points in time^31,32^.

Smoking causes mood swings that can be conducive to the onset of anxiety^33^. Munafo et al.^34^ found an association between depressive symptoms and smoking, with a large proportion of women facing both depression and anxiety, and these two conditions are often interrelated^28-30^.

Mothers whose babies had a problem were more likely to suffer from anxiety, which is in line with other studies^35,36^. Support from healthcare professionals is crucial, especially if newborns are admitted to hospital. Indeed, the proper treatment by health professionals involved in postpartum care emerged as a protective factor against anxiety. Zhou et al.^37^ found in their systematic review with meta-analysis where they identified a total of 11 studies and a sample of 2424 women from six different countries, that this is crucial for positive effects on maternal mental health. Women’s experiences would improve if they were given the opportunity to establish a trusting relationship with health professionals^38^.

Poor partner relationships and even abuse have been shown to be a variable recurrently associated with the onset of anxiety, postpartum depression, and even perinatal suicide^11,17^ which coincides with our findings. Women consider the emotional and practical support of their partners to be fundamental to mitigating anxiety^38^ although there are researchers who found opposite results, with the role of the partner being of little importance and not being associated with the presence of anxiety^39^.

If mothers had experienced a mental health problem during their lifetime, they were more likely to suffer from anxiety, which is in line with other authors^40^.

The mother–baby bond acts as a protective factor for the development of anxiety during the postpartum period, something that has been found by other authors^41^. Figueiredo and Costa^42^ found that anxiety is associated with poorer bonding, which may produce strong negative emotions toward the baby and less emotional involvement with the baby. This can have implications for the cognitive and physiological development of the newborn at an estimated cost of almost £6 billion^43^.

Several authors found that socioeconomic status plays an important role in increasing the risk of anxiety in women who do not have the resources to meet their financial needs^11,44,45^. This is in line with our findings that having a moderate/high monthly income protects against the possibility of postpartum anxiety.

### Strengths and limitations

The cross-sectional nature of this study limits the ability to draw causal inferences between the identified risk factors and postpartum anxiety. However, this limitation also presents an opportunity for further research in this area. While many of the assessment tools employed were self-reported questionnaires, the presence of a trained midwife or healthcare professional during the completion of these questionnaires helped to minimize potential bias. Consideration should be given to the temporal context of data collection and its potential impact on mental health outcomes in future studies. Enhancing the diversity of samples, including participants from various regions or countries, could improve the reliability of the findings. The questions were designed to be comprehensible across varying educational levels, thereby reducing the likelihood of classification bias. Furthermore, the sample was representative of the target population, with the mean age of participants aligning with the national mean maternal age in the country where the research was conducted. Although recall bias was a consideration, it is unlikely to have significantly affected the results, as the information solicited pertained to recent and salient experiences that would be difficult for participants to forget. One of the primary strengths of the study lies in the use of tools that have been specifically validated and adapted for the population studied, thereby enhancing the relevance and applicability of the findings.

## CONCLUSIONS

The present study shows that a high percentage of women experience anxiety, with a notable prevalence of mild anxiety, and a significant number facing moderate and severe levels. Factors such as a history of mental health problems, smoking, and baby-related complications increase the risk of developing anxiety, while strong bonding, adequate care from health professionals, and a stable economic situation seem to offer greater protection against this condition. Knowledge of risk factors will help health professionals providing care to postpartum women to recognize warning signs, enabling early detection and care.

## Data Availability

The data supporting this research are available from the authors on reasonable request.
